# On the practical, ethical, and legal necessity of clinical Artificial Intelligence explainability: an examination of key arguments

**DOI:** 10.1186/s12911-025-02891-2

**Published:** 2025-03-05

**Authors:** Justin Blackman, Richard Veerapen

**Affiliations:** 1 Island Medical Program, Faculty of Medicine, University of British Columbia, University of Victoria, Victoria, BC Canada; 2 School of Health Information Science, University of Victoria, Victoria, BC Canada

**Keywords:** Artificial Intelligence, Explainability, Black Boxes, Autonomy, Informed consent, Trust, Legal, Debate, Decision-support Systems

## Abstract

The necessity for explainability of artificial intelligence technologies in medical applications has been widely discussed and heavily debated within the literature. This paper comprises a systematized review of the arguments supporting and opposing this purported necessity. Both sides of the debate within the literature are quoted to synthesize discourse on common recurring themes and subsequently critically analyze and respond to it. While the use of *autonomous* black box algorithms is compellingly discouraged, the same cannot be said for the whole of medical artificial intelligence technologies that lack explainability. We contribute novel comparisons of unexplainable clinical artificial intelligence tools, diagnosis of idiopathy, and diagnoses by exclusion, to analyze implications on patient autonomy and informed consent. Applying a novel approach using comparisons with clinical practice guidelines, we contest the claim that lack of explainability compromises clinician due diligence and undermines epistemological responsibility. We find it problematic that many arguments in favour of the practical, ethical, or legal necessity of clinical artificial intelligence explainability conflate the use of unexplainable AI with automated decision making, or equate the use of clinical artificial intelligence with the *exclusive* use of clinical artificial intelligence.

## Background

Artificial intelligence (“AI”) is an increasingly popular field of research with numerous clinical applications identified and many decision support tools in various stages of development at present. AI demonstrably outperforms medical practitioners at specific tasks [1–3] and its continued performance improvement and integration into clinical practice are all but certain. However, integrating AI tools into clinical practice is not straightforward as it has opened the door to opined ethical dilemmas and unknown legal implications.

One aspect of clinical AI (“cAI”) that has been heavily developed and debated is the attribute of *explainability*, which is often defined to the effect of operating with sufficient transparency in reasoning and/or post hoc analysis as to allow the user an understanding of “why predictions are made, or how model parameters capture underlying biological mechanisms” [4]. The prototypical example of a cAI explanation is the use of heat maps in radiological image analysis, whereby salient features of an analyzed image are colour-coded based on the importance assigned to them by the AI. The exact form of a cAI explanation depends on the type of data analyzed and the context of use and can include methods like highlighting salient text or tabulating parameters that are within relevant limits [5].

Ethicists, clinicians, and computer scientists use the term *explainability* to signify various related concepts and thus there is no universal definition of the term. What is meant by *explainability* in this paper is perhaps most clearly communicated by a definition of its absence: “whenever the reasons why an AI decision-maker has arrived at its decision are not currently understandable to the patient or those involved in the patient’s care because the system itself is not understandable to either of these agents” [6]. AI tools that are not *explainable* are herein referred to as *black boxes*, as is typical of the literature. Including *understandability* in the intended meaning of *explainability* ‌is purposeful and nuanced as, strictly speaking, *explainability* of a programmatic AI tool is indefeasible [7] and technically, by virtue of its programmatic nature, even the most complex of *“*un*explainable”* AI algorithms can have its inner workings completely described. However, this analysis might be so involved and unwieldy that it is effectively unintelligible to mere humans. Related terms such as *transparency* [8], *intelligibility* [9], *interpretability* [10], and *explicability* [11] have all been used with varying degrees of conflation with the meaning of *explainability* intended herein; luckily, use of these related terms is as a rule accompanied by the term “explainable”, and often also includes the increasingly popular initialism xAI.

It has been postulated that e*xplainability* is necessary to maintain medical decision making accountability and to mitigate algorithmic biases. However, a recent systematic review concluded that there is no definitive agreement on the requirement of *explainability* in the literature [12]. The prohibitive development and/or performance costs make implementation of *explainability* challenging, especially in highly advanced deep-learning techniques that intrinsically cannot achieve *explainability* [13]. It is reasonable therefore, to question to what extent, if any, must we pursue AI *explainability* in medicine.

Many authors have put forth arguments for and against such necessity with practical, ethical, and legal bases and in doing so have identified several issues that significantly impact patient care. Several key questions remain to be answered; to what extent must a clinician understand the functioning of their diagnostic tools? how can a patient provide informed consent if they cannot understand how a diagnosis was provided? what if a black box AI tool is wrong, “*Quis custodiet ipsos custodes?”* [Who will watch the watchmen?] [14].

By synthesizing the points and counterpoints found within the literature, this systematized [15] review summarizes and responds to the arguments presented to the question: what *are the practical*,* ethical*,* and legal necessities of explainability in clinical artificial intelligence tools*? While previous reviews have examined *explainability* of AI in general [16], and other authors have provided narrative summaries of the cAI *explainability* debate [17], this review systematically illuminates the back-and-forth of argumentation within the literature vis-à-vis the necessity of cAI *explainability*. This paper also presents a novel critique within the discussion.

## Methods

**Fig. 1:**
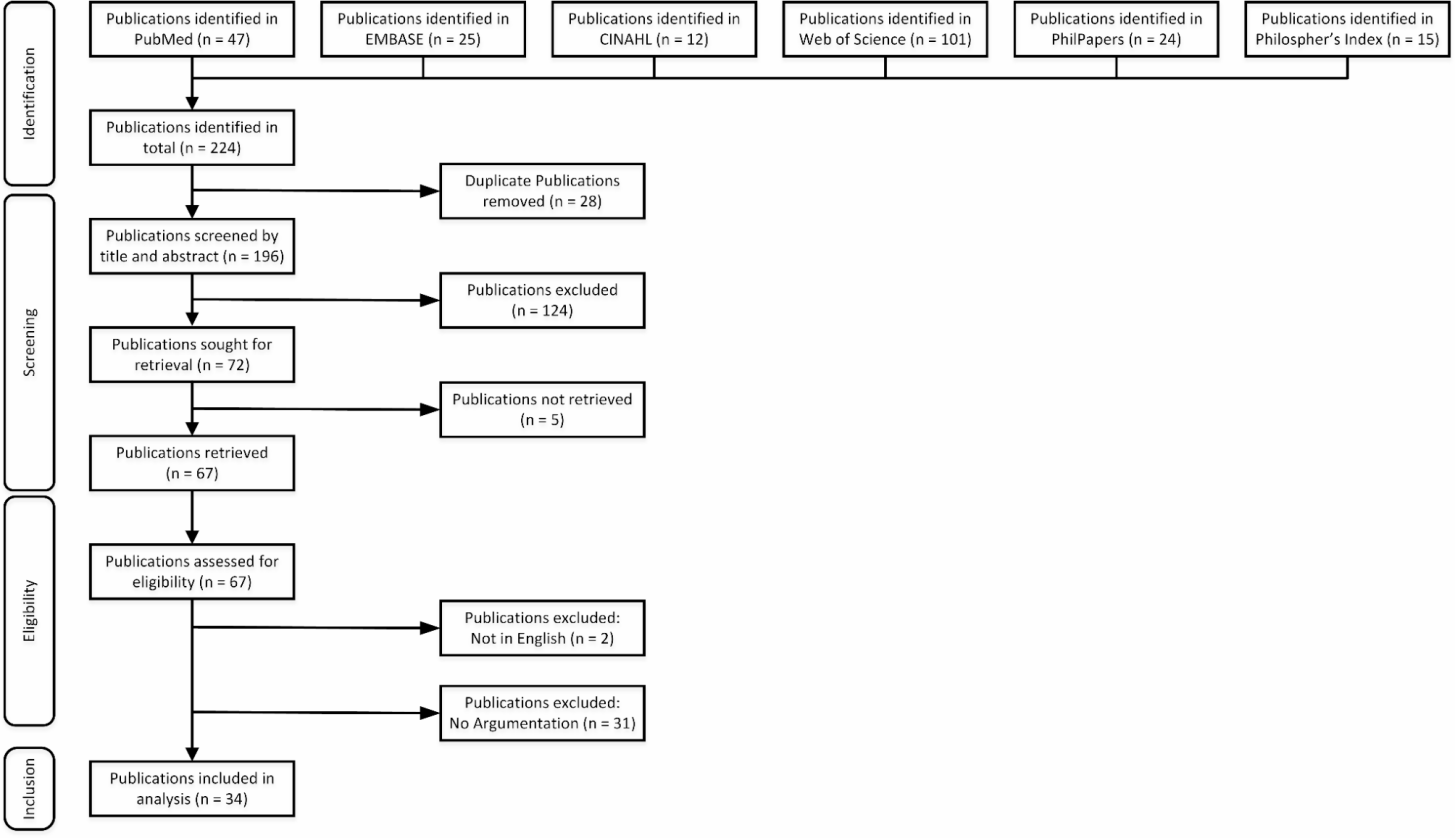
PRISMA flow diagram [18] of this review

Six cross-sectional databases were included in this review, each with its own emphasis on clinical sciences, technology, and philosophy: PubMed, EMBASE, CINAHL, Web of Science, PhilPapers, and Philosopher’s Index. The results of the search and screening strategy are summarized in Fig. 1. The database searches were conducted on May 1st, 2024, using dedicated search strings as provided in Table 1; the searches were not constrained by publication date or any other measure of time. Notably the search strings included the terms “interpretable”, “interpretability”, “explicable”, “explicability”, and “illustratable” in order to capture the essence of the intended search terms “explainable” and “explainability” (which were also included), in light of the aforementioned terminology discord throughout the literature. Inclusion and exclusion criteria, set out in Table 2, were determined prior to conducting the search. A paper was considered to *provide argumentation* only if it included some line of reasoning with premises justifying the associated claim; merely stating a perceived advantage or disadvantage of *explainable* cAI would not be sufficient to warrant inclusion, for example.

**Table 1:** Search strings

PubMed	(“explainable” OR “explainability” OR “interpretable” OR “interpretability” OR “explicable” OR “explicability” OR “illustratable”) AND ((“Information Science”[MeSH Terms]) OR (“Medical Informatics Applications”[MeSH Terms])) AND (jurisprudence[MeSH Terms] OR ethics[MeSH Terms]) Full Text[Filter]
EMBASE	(explainable OR explainability OR interpretable OR interpretability OR explicable OR explicability OR illustratable) AND (“artificial intelligence” OR AI OR “machine learning” OR informatics) AND (jurisprudence OR law OR statute OR precedent OR legal OR ethics OR ethical OR bioethics OR moral)
CINAHL	(explainable OR explainability OR interpretable OR interpretability OR explicable OR explicability OR illustratable) AND (“artificial intelligence” OR AI OR “machine learning” OR informatics) AND (jurisprudence OR law OR statute OR precedent OR legal OR ethics OR ethical OR bioethics OR moral)
Web of Science	(ALL=((explainable OR explainability OR interpretable OR interpretability OR explicable OR explicability OR illustratable) AND (“artificial intelligence” OR AI OR “machine learning” OR informatics) AND (jurisprudence OR law OR statute OR precedent OR legal OR ethics OR ethical OR bioethics OR moral)) AND TI=(medicine OR medical OR clinical OR healthcare OR health-care OR “health care”))
PhilPapers	(explainable| interpretable| interpretability) & (“artificial intelligence”| AI| “machine learning”| informatics) & (medical| clinical)
Philosopher’s Index	mainsubject.Exact ((“medical information” OR “medical practice” OR “medical profession” OR “medical” OR “medical philosophy” OR “medical care” OR “medical technology” OR “medical judgment” OR “medical law” OR “medical data” OR “medical professionals” OR “medical ethics” OR “medical knowledge”) AND (“artificial intelligence” OR “information technology” OR “generative artificial intelligence” OR “machine learning”))

**Table 2:** Inclusion and exclusion criteria

*Inclusion criteria*	*Exclusion criteria*
● Articles, Editorials, Reports, or Commentary on the ethical and/or legal necessity of *explainability* in clinical implementations of AI ● Published in a peer-reviewed journal	● Does not provide argumentation for either claims or premises ● Not in English

Retrieved publications were subjected to a systematic screening process. Initially, duplicates were identified and removed. Titles and abstracts were then reviewed to ensure relevance to practical, ethical, and/or legal considerations on the *explainability* of cAI. Full-text screening for inclusion and exclusion criteria was subsequently performed.

Coding of the screened publications was conducted using a grounded theory methodology implemented in ATLAS.ti. Each publication was examined line-by-line during this process, with relevant arguments or examples assigned an initial descriptive code based on their content. Related code instances occurring across publications were iteratively organized and refined. The resulting categorizations were then abstracted into the identified themes of argumentation.

## Results and Discussion

**Fig. 2:**
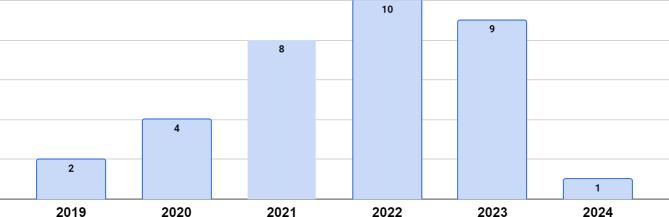
Publication years of the works included in this review

**Fig. 3:**
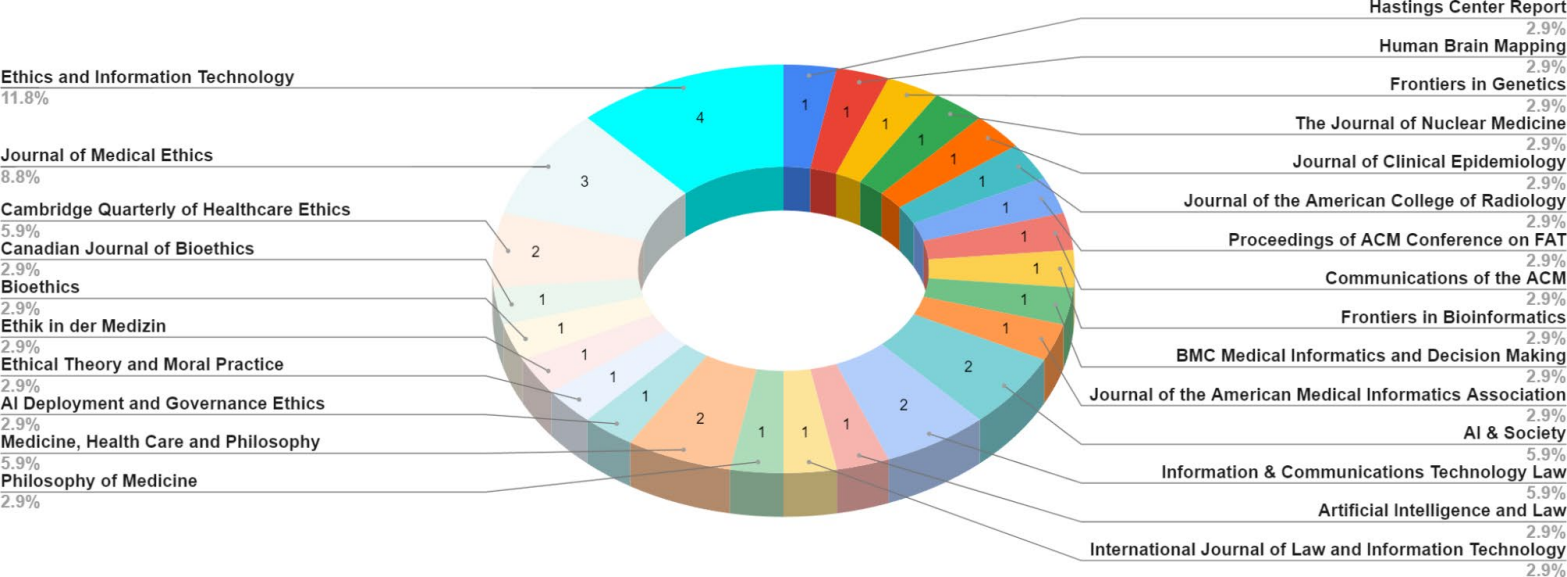
Publishing journals of the works included in this review

This review analyzed thirty-four publications, with the full list of included works (following retrieval and screening) provided in the Appendix to differentiate them from the other references in the study. The distribution of publication dates for the works included in this analysis are provided in Fig. 2, and the journals of publication for the included works are noted in Fig. 3; the screened works span 6 years and 25 journals.

**Fig. 4:**
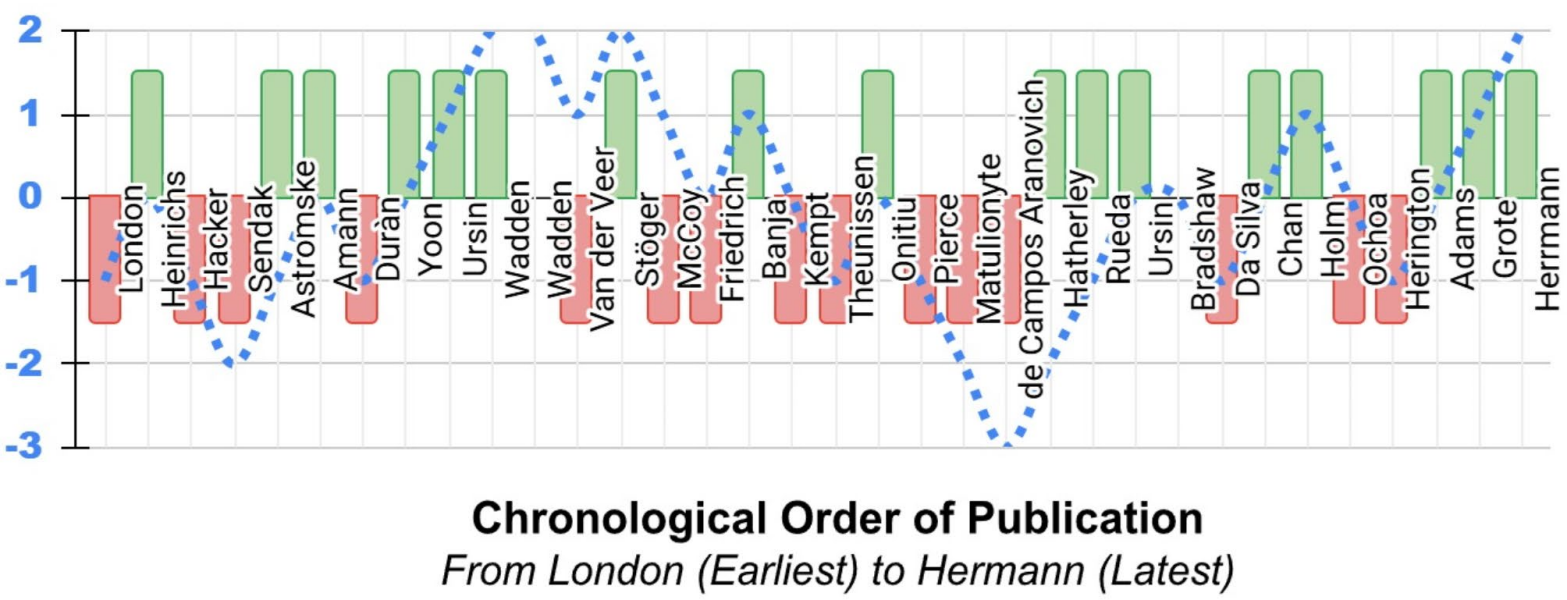
Sentiment analysis of the works included in this review

Figure 4 depicts the literature’s broad sentiment on the necessity of cAI *explainability* by indicating whether each publication included in this review supports (positive upgoing green bars) or opposes (negative downgoing red bars) this necessity, and provides a running tally of the votes in favour less those opposed over time (dotted blue line). There is clearly no consensus nor temporal trend in the sentiment among the analyzed works.

Two opposing authors were repeatedly mentioned, emerging as figureheads on either side of the *explainability* debate. London posited the value of accuracy above *explainability* and argued that this approach to cAI is the only one consistent with the goals of existing evidence-based medicine practices [10]. Alternatively, Floridi postulated *explainability* was so necessary that it must be incorporated as a fifth biomedical ethical principle for cAI [11] in addition to the original Beauchamp & Childress principles of autonomy, non-maleficence, beneficence, and justice [19]. These works were so influential as to lead to the establishment of reliabilist and principlist camps that clearly divide the literature.

Nine themes of argumentation were identified within the arguments put forth to support or oppose the necessity of *explainability* of cAI; these themes and the corresponding conflicts of principles, values, or claims are put forth in Table 3.

**Table 3:** Themes of argumentation identified within reviewed works

*Theme* (Relevant Section)	*Conflict of Principles*,* Values*,* or Claims*
Epistemological Priority *(See* Sect. 4.1*)*	Theoretical transparency (normative clarity) vs. Empirical validation (pragmatic outcomes)
Bias-Variance Performance Dilemma [20] *(See* Sect. 4.2*)*	Pragmatic outcomes through generalizability and mitigation of bias vs. Pragmatic outcomes through accuracy and efficiency
Autonomy [19] and Informed Consent *(See* Sect. 4.3*)*	Understanding of underlying mechanistic processes (epistemic requirement) vs. Understanding of potential benefits and harms (ethical imperative)
Justice [19] *(See* Sect. 4.4*)*	Critique of reasoning (normative dimension) vs. Critique of process (procedural dimension)
Patient and Practitioner Trust in Technology *(See* Sect. 4.5*)*	Trust through transparency in outcome (normative claim) vs. Trust through transparency in development (procedural requirement)
Due Diligence and Liability *(See* Sect. 4.6*)*	Decision value of a process (normative claim) vs. Decision value of a result (descriptive claim)
Legal Statute *(See* Sect. 4.7*)*	Right to explanation (legal obligation) vs. Suggestion for explanations (legal best practice)
Achievability *(See* Sect. 4.8*)*	Sufficiency of idealization (epistemological claim) vs. Real-world complexity (pragmatic challenge)
Scientific Discovery *(See* Sect. 4.9*)*	Potential for new knowledge (empirical benefit) vs. Risk of false mechanistic reasoning (epistemological caution)

The discussion found within the analyzed publications for each theme of argumentation is summarized in the following subsections. Representative quotations in the following Tables 4, 5, 6, 7, 8, 9, 10, 11 and 12 (one in each subsection) are provided to approximate a discourse of how the authors would respond to each other’s arguments. The rows of each **Table** are organized to present synthesized argument-rebuttal pairs. The first column presents a synthesized argument which is labelled as either in favour or opposed to the necessity of *explainability* of cAI; a synthesized rebuttal to the argument is then provided in the second column. A summary of the discourse for each topic is stated, followed by our subsequent analysis, after each **Table**.

### AI *Explainability* and Epistemological Priority

**Table 4:** Discourse on AI *explainability* and epistemological priority

*Arguments*	*Rebuttals*
***OPPOSED*** ** to the necessity of** *** explainability*** ** of cAI**	
“Although medicine is one of the oldest productive sciences, its knowledge of underlying causal systems is in its infancy; the pathophysiology of disease is often uncertain, and the mechanisms through which interventions work is either not known or not well understood. As a result, decisions that are atheoretic, associationist, and opaque are commonplace in medicine. […] As counterintuitive and unappealing as it may be, the opacity, independence from an explicit domain model, and lack of causal insight associated with some of the most powerful machine learning approaches are not radically different from routine aspects of medical decision-making […] the practical findings from rigorous empirical testing are frequently more reliable and reflective of causal relationships than the theoretical claims that purport to ground and explain them” [10]	“there are differences between the transferability of drug [randomized controlled trails (RCT)]s and RCTs involving ML models […] effect sizes from drug RCTs are often claimed to be transferrable to nonexperimental settings. The same does not apply to RCTs for ML models, given that distribution shifts can severely impact the model’s performance, even if it is used in another hospital in the same city [… for example] poor lighting in a hospital in Thailand led to many ungradable images for an ML model, used to detect diabetic retinopathy, while the same ML model surpassed expert ophthalmologists during training” [21]
***OPPOSED*** ** to the necessity of** *** explainability*** ** of cAI**	
“medicine has a long and ongoing history of harnessing technologies (in a broad sense, including pharmaceuticals, procedures, and diagnostic aids) for which physicians lack clear mechanistic explanations […] From acetaminophen to metformin, or many antidepressants and mood stabilizers, numerous medications are prescribed regularly—and to great effect—despite the fact that their mechanisms of action are partially or even entirely unclear. Even certain surgical procedures, such as gastric bypass for obesity, are performed despite their mechanisms of action not being fully understood” [8]	“the comparison with lithium’s clinical trials here is inapt. […] while physicians would not know the weighted factors in the AI decision, doctors would know them in their decision to prescribe lithium. While the weighted factors by themselves likely do not provide an adequate explanation of the reason for the decision, they could be a first step towards one […] e.g., if my patient’s age or ethnicity were different such that it was very unrepresented by the subjects of the clinical trials, then I would not prescribe lithium” [22]
***IN FAVOUR*** ** of the necessity of** *** explainability*** ** of cAI**	
“The central aim of [evidence-based medicine] is ‘to ensure that decision making in health care incorporates the best available evidence.’ Importantly, the incorporation of the best evidence is supposed to be judicious ‘taking into account both clinical expertise and the needs and wishes of individual patients.’ […] Importantly, clinical expertise involves both interpretation and appropriate application of the evidence in the circumstances.” [23]	“Medical knowledge is typically incomplete. For many diseases, no known biomarkers exist and, given the complexity of physiological processes, it can be difficult to assess whether the relationship between variables is causally relevant or spurious” [21] “mechanistic reasoning has been considered lesser than correlative or statistical reasoning in hierarchies of evidence” [8]

Those opposing the necessity of *explainability* of cAI contend that opaque decision-making aligns with established evidence-based medicine (“EBM”) practices whereby mechanisms of action for treatments can remain unknown and the treatments are nonetheless used, if proven effective. Through examples, they argue that medicine routinely relies solely on empirical outcomes when lacking mechanistic understanding. On the other hand, advocates for *explainability* of cAI argue that EBM requires a critical appraisal of results that can only be achieved through the interpretability of the studies that led to them.

Based on the arguments raised, *explainability* must be demanded of cAI only if such an explanation is necessary to practice EBM. EBM has been defined as “the conscientious, explicit, and judicious use of current best evidence in making decisions about the care of individual patients” [24] and the steps to apply EBM have been stated as defining a clinically relevant question, searching for the best evidence, critically appraising the evidence, applying the evidence, and evaluating the performance of EBM [24, 25, 26]. ‌The crux of the matter becomes whether a cAI output can be critically appraised without a provided explanation; arguably the development and testing of a *black box* AI can be critically appraised analogously to the methods of a treatment trial, and thus both are consistent with EBM practices in spite of a mechanistic explanation. In the same way that one author argues against the use of *black boxes* by stating that they would need to examine if their “patient’s age or ethnicity were different such that it was very unrepresented by the subjects of the clinical trials” [22], so too can a clinician compare their patient’s demographic data with that of the training data used for a *black box*. To our mind, the concerns raised regarding lack of generalizability of an algorithm [21] speak to the quality of the algorithm and its training data and are identifiable based on transparent development practices rather than output *explainability* [17]; furthermore AI *explainability* does not automatically imply generalizability, as a seemingly rational explanation can still produce incorrect determinations.

### Performance and Bias-Variance Tradeoff of AI *Explainability*

**Table 5:** Discourse on the performance and bias-variance tradeoff of AI *explainability*

*Arguments*	*Rebuttals*
***OPPOSED*** ** to the necessity of** *** explainability*** ** of cAI**	
“the use of tests that are less sensitive (that is, less likely to detect true cases of a condition), less specific (less likely to exclude only false cases), or less precise (with less likelihood that a positive test result correlates with having the condition) than available alternatives can result in avoidable morbidity and mortality on the part of patients. Any preference for less accurate models—whether computational systems or human decision-makers—carries risks to patient health and welfare.” [10] “Trade-offs may arise between accuracy and explainability, as reducing opacity may motivate the use of more simplistic models, or the evaluation of smaller and more comprehensible pools of data” [8]	“The case for accuracy appears to erroneously assume a necessary causal link between technical accuracy and improved downstream patient health outcomes. While diagnostic and predictive accuracies are certainly important for the improvement of patient health outcomes, they are far from sufficient. […] One reason for this gap comes from the fact that human users do not respond to the outputs of algorithmic systems in the same way that we respond to our own judgments and intuitions, nor even to the recommendations of other human beings […] Medical AI systems need to be understood as intervening in care contexts that consist of an existing network of sociotechnical relations, rather than as mere technical ‘additions’ to existing clinical decision-making procedures” [27]
***IN FAVOUR*** ** of the necessity of** *** explainability*** ** of cAI**	
“Rudin denies that the predictive performance of deep learning models is necessarily more powerful than that of interpretable models. […] many of the seemingly most powerful models achieve their predictive performance by overfitting to the benchmark data […] by constraining the model architecture, or by incorporating domain knowledge, […] the interpretable model promises more stable performance across different settings and may lead to better real-world outcomes” [21]	**“**if both the predictions and the explanations can be wrong, this adds another avenue for an AI system to err.” [28]
***IN FAVOUR*** ** of the necessity of** *** explainability*** ** of cAI**	
“Ingrained biases within the data sets and mathematical formulae that train ML algorithms present a pernicious and potentially far-reaching threat to justice, which might remain undetected if [explainability] is not pursued.” [29] “explainable AI would allow clinicians to identify errors made by AI, override incorrect decisions and thus avoid harm, as well as help improve AI technology” [30] “A prototypical example of this is a [healthcare machine learning] model which erroneously identified asthma as a protective factor against pneumonia severity, when in reality the ‘protective effect’ was a manifestation of the aggressive use of intensive care for asthmatic patients” [8]	“the ascription of bias in this example presupposes that the goal of the decision model is to optimize the allocation of medical resources against a baseline risk of death that is independent of current medical practice. But insofar as the training data reflect the probability of death given standard medical practice, this is probably an inappropriate expectation for many patients, not just for asthmatics. […] If given more comprehensive information about treatments administered to individual patients, even a simple system would learn that, without ICU admis- sion, asthma puts a patient at high probability of death.” [10]
***IN FAVOUR *** **of the necessity of ** ***explainability*** ** of cAI**	
“Explainability enables the resolution of disagreement between an AI system and human experts, no matter on whose side the error in judgment is situated.” [31]	“Claims that a human-in-the-loop must confirm AI decisions are […] largely orthogonal to present debates. Debates about AI explainability and the need for a human-in-the-loop are analytically distinct” [32]

Proponents of the necessity of *explainability* of cAI suggest that *explainable* algorithms exhibit superior generalizability by incorporating domain knowledge, avoiding overfitting, and finding balance in the bias-variance performance tradeoff [20]. They also argue that that explainable models also enable clinicians to identify errors and override incorrect decisions. Opponents assert that prioritizing explainability over raw performance necessarily results in worse patient outcomes by the very nature of the misprioritization. Critics also point out that both *explainable* cAI and *black boxes* can incorporate human-in-the-loop decision frameworks.

Our analysis of these arguments is grounded in framing the discussion through the lens of mathematical optimization. Machine learning algorithms are those that “automatically alter or adapt their architecture through repetition (i.e., experience) so that they become better and better at achieving the desired task” [33]. Within the conceptual set of all such possible algorithms that can achieve a desired task, some portion of the set will be *explainable* and the rest will not. By limiting our scope of allowable algorithms only to those that possess *explainability*, we restrict our options to a subset of the original domain within which the best performing algorithm may or may not reside. Thus while a particular algorithm with *explainability* may outperform a particular algorithm without it, as a class, algorithms with *explainability* can at best achieve non-inferiority relative to the class of algorithms without this domain constraint. This mathematical truth holds despite accusations of the overfitting of particular deep learning models and improved generalizability of particular models with *explainability* [21] as these observations speak to the implementations of particular instantiations of algorithms rather than to global considerations of the algorithm classes in whole. When considering classes of technology, and not any one implementation in particular, the demand for *explainability* must accompany a non-negative performance cost. *Explainability* is only one of many possible methods to control for overfitting; cross-validation techniques [34], where a subset of the available data is withheld from algorithm training and used for algorithm testing, are ubiquitously used to avoid overfitting during development without the use of prediction explanations. Furthermore, the prevailing assumption that clinicians will correct errors made by AI when given output explanations is challenged by the recent findings that clinicians “struggle to consistently distinguish between accurate and inaccurate AI predictions and can be misled by inaccurate AI predictions” [35].

### AI *Explainability*, Autonomy, and Informed Consent

**Table 6:** Discourse on AI *explainability*, the ethical principle of autonomy [19], and informed consent

*Arguments*	*Rebuttals*
***OPPOSED*** ** to the necessity of** *** explainability*** ** of cAI**	
“informed consent has never required a mechanistic understanding of an intervention, only its risks and benefits” [28] “The package insert indicating potential side effects is an illustrative example of the information required for an acceptable interpretability of patients in medical decision- making, and under consideration relative explainability, arguments of why AI should provide much more than presume a double-standard” [36]	“Treating patients with effectiveness and respect for their dignity and autonomy requires being able to explain medical diagnosis or treatment recommendation. […] The physician being able to explain the diagnosis can stave off denial on the part of the patient and increase the chances of effective treatment. […] The physician being able to explain to the patient how the upsetting diagnosis was arrived upon could give them an element of personal dignity, because the patient would at least gain some sense of understanding of why they received it.” [22] “Health professionals would be failing to respect patients as autonomous agents if they do not recognize them as agents capable of receiving and processing the information that affects them. A communicative process that truly recognizes others as autonomous agents requires a dialogue seeking mutual understanding.” [37]
***IN FAVOUR*** ** of the necessity of** *** explainability*** ** of cAI**	
“contemporary legal issues concerning informed consent of the patients focus mostly on the scope of the information that must be sufficiently provided before the patient has to decide which healthcare services or providers to choose. Legal scrutiny shifted on whether consent given by the patient was supported with enough information to make competent decisions before consenting for certain medical treatments.” [9] “For obtaining informed consent for diagnostic procedures or interventions the law requires individual and comprehensive information about and understanding of these processes. In the case of AI-based decision support, the underlying processes and algorithms have therefore to be explained to the individual patient” [31]	“It is unclear, however, whether principles of informed consent require clinicians to explain to patients the precise causal pathway between diseases and diagnostic test” [38] “Extant norms do not require explanations of the mechanisms by which options will work that would bar lithium prescriptions [for example]” [32] “patients may need certain information about AI technology, like any other technologies applied in the healthcare sector, the information they would require would fall under the ‘transparency’ concept […], rather than an explainability concept” [17]
***IN FAVOUR*** ** of the necessity of** *** explainability*** ** of cAI**	
“full autonomy can only be achieved if the patient is presented with a range of meaningful options to choose from. In this respect, patients’ opportunities to exert their autonomy regarding medical procedures get reduced as opaque AI becomes more central to medical decision making” [31]	“[you] point to the need for a regulatory process that ensures AI and providers provide enough information to patients so they can make decisions and that both respect patient choices. AI introduces additional loci for recommendations that complicates the informed consent process. However, the need to respect patient preferences is orthogonal to explainability questions. And the level of information patients require to make decisions once again does not support strong explainability requirements” [32]
***IN FAVOUR*** ** of the necessity of** *** explainability*** ** of cAI**	
“The health professional and the patient should not only understand the basic functionality of the AI system, but it is the grasp of the model’s feature importance, being relevant for the doctor and the patient when deciding on further recommendations for treatment. In this respect, a healthcare professional needs to respect patient autonomy, amongst others” [39]	“an appropriate and useful explanation need not involve post hoc explanations at all, since explaining other features of the machine—how it works, what contexts it works in, how it was trained—may be sufficient for justifying its usage […] The takeaway is that it is not necessarily the case that post hoc explanations increase epistemic grounds for relying on the machine in the first place. And it is also not necessarily the case that a lack of post hoc explanation makes a machine untrustworthy.” [40]
***IN FAVOUR*** ** of the necessity of** *** explainability*** ** of cAI**	
“a patient’s appreciation of risk is consequential to the system’s performance metrics. However, patient autonomy is based on the individual’s action to increase a patient’s wellbeing, as well as enabling patient to act on the beliefs and values they hold. What this shows is that a system’s probabilistic judgements become the defining feature for the individual to evaluate treatment recommendations including a patient’s values. Once probabilistic judgements become prescriptions, then patient autonomy is negated” [39]	“These worries are understandable and would indeed be worrisome if black box algorithms would automatise decision making, without humans in the loop […] Note though that these problems are not caused by the opaqueness of the underlying algorithm but by the lack of choice provided.” [41]

Opponents of the necessity of *explainability* of cAI argue that informed consent has never required a mechanistic understanding of a pathology or the correction implemented by a therapy; they emphasize that mechanistic understandings do not exist for many common medications, and that medication package inserts only describe possible adverse reactions and side-effects. In contrast, proponents suggest that an individual must be able to evaluate probabilistic judgements in regards to their care in order to enact autonomy, which requires an understanding of feature importance within a cAI. They also argue that the law sets out minimum standards of information that must be provided to patients that necessitate *explainability*.

In our view, issues of informed consent arise with cAI when either: (a) a patient has been diagnosed with the support of cAI and subsequently is suggested a treatment considering the diagnosis, or (b) when a cAI tool has recommended a course of treatment for a patient with a prior diagnosis. A *black box* cAI-assisted diagnosis is no more obstructive to informed consent for subsequent treatment than a diagnosis of idiopathy, or one of exclusion, since with the former no clear explanation of cause exists and with the latter no definitive diagnostic methodology exists. Yet, the literature is silent on the issue of informed consent given idiopathic diagnosis or diagnosis of exclusion; while this may be a shortcoming of the literature, it is more likely indicative of a double standard [42] raised in the argument against *black box* cAI. In the case of a cAI-generated recommendation of a course of treatment for a patient, we can delineate two possibilities again: either the clinician is using the cAI alongside other existing knowledge and frameworks in order to devise a treatment plan, or the clinician has no other information on which to base their selection of treatment. If the former, though an explanation might facilitate the clinician’s assessment and incorporation of the cAI output, the use of a *black box* would not prevent them from relying on the cAI output or explaining their diagnostic rationale to the patient. If the latter, the explanation from the cAI is moot since the clinician could not assess the explanation and would therefore solely rely on empirical evidence justifying use of the cAI as if it were a *black box*, anyway. Thus, mechanistic reasoning is neither presently consistently available, nor necessary to respect autonomy and achieve informed consent.

### AI *Explainability* and Justice

**Table 7:** Discourse on AI *explainability* and the ethical principle of justice [19]

*Arguments*	*Rebuttals*
***IN FAVOUR*** ** of the necessity of** *** explainability*** ** of cAI**	
“[Explainability is] necessitated by the principle of justice, which requires patients to be allowed to understand and appeal against healthcare outcomes on a fair and equal basis” [43] “patients may claim that they are being discriminated against when they are not given similar opportunities to clear their doubts compared to others.” [37]	“Patients have traditionally had a right to know what technology will be used in diagnostic processes, what are the benefits and risks, as well as financial implications of technology, which could be defined as ‘transparency’ around technology, but not how exactly technology functions (explainability). The same standards should apply with relation to AI technologies.” [30]
***OPPOSED*** ** to the necessity of** *** explainability*** ** of cAI**	
“public reason standards required for health justice never necessitated full transparency in how medical tools work. They required good reasons for decisions and opportunities to challenge them, which can be and are often provided without tools being explainable. Legal mechanisms for evaluating AI tools present numerous opportunities to assess performance, costs, and reasons for adoption and a framework for assessing accuracy and justifiability” [32]	“whereas accuracy is mainly relevant from outcome-oriented stances, explainability is a requirement for procedural fairness accounts […] One area where the inexplicability of AI is of particular concern: the allocation of scarce medical resources […] Accountability for reasonableness—which remarks that fair processes need transparency, publicity on rationales, and open mechanisms to revise the decisions— can be applied to XAI and distributive justice in medicine” [44]
***IN FAVOUR*** ** of the necessity of** *** explainability*** ** of cAI**	
“The medical records of some of the most vulnerable groups, especially from technologically underdeveloped territories, might be poorly collected or digitized, thus resulting in sample size disparity. Therefore, available raw data may reflect and expand existing bias and, in turn, unfairly affect members of protected groups based on sensitive categories like gender, race, age, sexual orientation, ability, or belief” [9] “The problem of bias, nevertheless, is not solved by simply trying to assess algorithmic performance across diverse demographics. Technology-centred solutions are limited when they neglect that biases are also a sociopolitical issue related to underlying health inequities in society. Biases can surreptitiously lead to favouring or disadvantaging particular social groups in contexts of historical discrimination, which can lead AI to reproduce societal prejudices and systemic inequalities, or even reinforce discriminatory practices. An opaque or unexplainable procedure prevents the verification of whether the decision is free from inappropriate considerations and unethical biases” [44]	

The major themes raised in this discussion are those of procedural fairness and distributive justice. Those in favour of the necessity of *explainability* of cAI argue that *black boxes* pose ethical concerns as they do not afford individuals the right to understand and appeal a decision process. They also suggest that *black boxes* necessarily predispose systems to a high risk of prejudice by virtue of their opaqueness. Those opposed argue that *black boxes* can be evaluated for systemic bias, and that the transparency required of cAI is what describes its development and validity, not reasoning.

Upon examining the preceding discussion, it becomes evident that proponents of *explainable* cAI often equate *black box* cAI with autonomous cAI. Yet *black boxes* can be implemented within a so-called human-in-the-loop [45] workflow; an allocation of healthcare resources augmented with the input of a *black box* cAI would maintain a patient’s right to understand and appeal the decision process used by the human in the loop. Data availability, bias, and prejudice disadvantaging particular social groups and contributing to further discriminatory systemic inequality by *black boxes* are extremely valid concerns. However, not only do these concerns equally affect *explainable* cAI (as the *explainability* of the algorithm has no effect on the quality of the data with which it is trained), they impact existing quotidian diagnostic tools [46, 47]. The required solution is identical for each of these technologies: commitment to continual improvement in health equity by all those involved in the development, use, and quality assurance of the technology.

### AI *Explainability* and Trust

**Table 8:** Discourse on AI *explainability* and clinician and/or patient trust

*Arguments*	*Rebuttals*
***IN FAVOUR*** ** of the necessity of** *** explainability*** ** of cAI**	
“how can we trust our health, let al.one our very lives, to decisions whose pathways are unknown and impenetrable? Indeed, without established trust, a patient may have little or no incentive to seek the advice of a physician or share sensitive clinical information, which is required by the artificial intelligence algorithms for diagnostic purposes” [9]	“explainability is an instrumental means of establishing and maintaining trust and control, but is not a critical end in and of itself” [8] “a mechanistic understanding of how an intervention works is not necessary for either trust or transparency” (Bradshaw T.J. et al., 2023)
***OPPOSED*** ** to the necessity of** *** explainability*** ** of cAI**	
“clinicians need transparency around the technology they use to ensure certain levels of trust. However, clinicians do not necessarily need an in-depth explanation of how each AI recommendation or outcome is generated, if they are comfortably satisfied that the technology is accurate and reliable, they being the most important factors in ensuring trustworthiness.” [17] “patients trust technology if their doctors recommend it. The concepts of trust and delegation are inherent to this market.” [30]	“Unfortunately, trust is not something that is so easily transferred. We can easily imagine a patient who trusts the professional in most circumstances but fails to trust them whenever they outsource part of the decision-making process to an AI system.” [48] “patients rely on the clinician’s ability to understand and convey […] explanations in a way that is accurate and understandable” [31]

Opponents of the necessity of *explainability* of cAI suggest that clinicians may satisfy themselves as to the development rigor and accuracy of *black boxes* without the need for explanations and by extension, patients can trust these technologies through the delegation inherent in relying on their clinician. Proponents of cAI *explainability* argue that clinicians and/or patients are justified in requiring an explanation regarding the determination made by a cAI in order to trust it.

We find points of contention with aspects of both sides of the argument. In our experience, patients vary widely in their preference for the amount of detail expected in the communication of their diagnosis and care plan, as is reported in the literature [49]. However, in no case would patients be reasonable in predicating their trust in their medical practitioner on the clinician’s ability to produce a perfectly accurate causal explanation for their illness or definitive diagnostic methodology; in fact, the public’s greater trust in accurate cAI systems over understandable ones has been demonstrated empirically [50]. In light of the existence of idiopathic illnesses and diagnoses by exclusion, the use of a *black box* does not seem so novel, nor therefore problematic, compared to present practices. The role of the clinician is in part to convey complex concepts to the patient, and so the patient is in part reliant on the clinician’s ability to achieve their own understanding. However, patients do not — and should not — completely delegate their determination of trust in a medical technology to their clinician, as was argued.

### AI *Explainability* and Liability

**Table 9:** Discourse on AI *explainability*, due diligence, and legal liability

*Arguments*	*Rebuttals*
***IN FAVOUR *** **of the necessity of ** ***explainability*** ** of cAI**	
“Opacity can be morally problematic in cases where a clinician violates due diligence, making treatment decisions based on an ML model’s prediction, while being in the dark about the underlying factors” [21] “by referring to their professional duties, doctors have an argument to insist that technology producers develop explicable AI-systems to adequately fulfill their responsibility of avoiding harm.” [37] “Given that clinical reasoning involves a range of tasks that cannot be deferred to AI systems, but must be undertaken by clinicians in collaboration with patients, it seems important to recognize clinicians as epistemologically responsible. […] given the epistemic responsibility of the clinicians curating the available information and best evidence about the patient, it only seems appropriate that they have the means to take on this responsibility. Being epistemically responsible, they must also be able to justify their reasoning and judgments. Requiring explanations of opaque AI output seems to support this.” [23]	“In daily life a sufficient explanation to a physician is an explanation that gives her enough justification to do or not do something […] accuracy should and does serve as a necessary and sufficient basis for responsible use of AI in [clinical decision support systems] by physicians.” [51]
***IN FAVOUR*** ** of the necessity of** *** explainability*** ** of cAI**	
“Physicians as domain experts should have access to the explanation why an algorithm reached a certain decision because the algorithm’s output justifies or contests their own decision” [52] “a physician consulting a black box AI can find herself in a rationally irresolvable situation if the AI output contradicts her diagnosis.” [53] “In the event of technology–physician disagreement, clinicians should be able to defend themselves” [54]	“if black box algorithms diagnose an illness and predicts which type of treatment would be most effective, the question what an acceptable and desirable way of acting is needs to be deliberated further based on this information, for which professional expertise and patient values are important” [41]
***IN FAVOUR *** **of the necessity of** *** explainability*** ** of cAI**	
“confronted with a black-box system, clinical decision support might not enhance the capabilities of physicians, but rather limit them. Here, physicians might be forced into “defensive medicine” where they dogmatically follow the output of the machine to avoid being questioned or held accountable” [31] “How can we then call for ultimate human responsibility, when we at the same time deprive a human operator from the epistemic means to live up to this responsibility?” [53]	“doctors are compelled, under negligence law, to exercise independent judgment and may disagree with the model […] not departing a wrong model prediction would breach the standard of care if, and only if, the reasons for departure were sufficiently obvious to a professional” [55]
***IN FAVOUR*** ** of the necessity of** *** explainability*** ** of cAI**	
“to provide patients with the most appropriate options to promote their health and wellbeing, physicians need to be able to use the full capabilities of the system. This implies that physicians have knowledge of the system beyond a robotic application in a certain clinical use case, allowing them to reflect on the system’s output.” [31]	“the use of the model should always only be part of a more comprehensive assessment, which includes and draws on medical experience” [55]
***IN FAVOUR*** ** of the necessity of** *** explainability*** ** of cAI**	
“If a clinician is subsequently sued because he or she accepted or rejected the model’s decision and the patient experienced resulting harm, a black box algorithm would compromise if not preclude the physician’s ability to defend him- or herself in court. […] If physician defendants allege that the model’s (unexplained) output decision seemed reasonable and therefore it was reasonable to follow it, they would not be able to counter the plaintiff’s rejoinder: that the patient’s injury is proof positive that the model’s output was not reasonable and that the physician defendant was negligent in failing to reject it. […] Physicians would therefore be exposed to significant liability for decisions that the technology precludes them from making but whose reasonability they cannot interrogate or, more importantly, justify.” [54]	“This claim is contestable, not only because physicians typically operate other technologies and machinery which they do not fully understand or cannot fully explain the inner working of (think of MRI scans, eg), yet they are sufficiently in control and understand enough of the workings to be considered responsible for operating these machines, including mistakes caused by these machines. […] for medical AI physicians can be responsible, in terms of accountability, for using these devices without fully knowing or understanding their inner workings […] responsibility can be ascribed to physicians when, under conditions of reliability, they were not morally justified in their actions.” [41]
***IN FAVOUR*** ** of the necessity of** *** explainability*** ** of cAI**	
“one might compare two scenarios in which an adverse patient event has occurred […] as a result of faulty ML ‘reasoning’ despite empirical validation […], with the only difference between the scenarios being the level of interpretability. […] One could then ask the question of whether the […] level of interpretability impacts the degree to which the attending physician is accountable for the adverse patient event. […] If this is the case, with the degree of interpretability constituting the only difference between these two scenarios, one must surely conclude that interpretability of ML models is relevant to accountability.” [29] “In case AI use results in harm and the court proceedings are started, the courts will need to understand how technology functions, how and why a particular outcome was generated, whether the technology is defective, and who should be held liable for the harm caused. Technical AI Explainability will be arguably important in determining and allocating liability.” [30]	“Instead of relying on technical explanations generated by XAI, court experts are likely to need access to various parts of the module, such as algorithmic parameters, training information, validation information and outcomes, clinical testing information, regulatory approval details etc. Experts might need to conduct an independent validation/audit of the system in order to determine whether it has a specific defect that caused harm and who is responsible for the defect. Thus, instead of technical explainability, they will require transparency around AI module […] the court experts will be invited to examine whether the AI development process met industry standards and legal regulations, and whether the AI manufacturer took all reasonable steps to avoid any harm and eliminate any possible errors/defects from software” [30] “On the issue of explainability, when determining whether there is a breach of duty by the clinician, it may not be directly determinative whether the clinician knew precisely how a particular AI device functioned and how it arrived at its decision. As indicated previously, clinicians often work with complex technology that they do not understand, whether fully, partly or at all, and rely upon their outputs. […] The focus is not on the clinician’s knowledge of the technology, but on their activities—whether they acted reasonably and with sufficient skill and care to prevent any possible harm.” [17]

Advocates for *explainable* cAI argue that *black boxes* undermine clinicians’ ability to fulfill ethical and legal responsibilities, as without an intelligible explanation they cannot evaluate the validity of cAI recommendations, or justify the decision to defy them. They suggest that *black boxes* force an untenable situation wherein clinicians are simultaneously liable for the shortcomings of cAI that they cannot interpret while also being incapable of justifying contradicting the cAI as there is no provided reasoning for them to refute. Opponents counter that reasonable judgment can be exercised in the absence of cAI explanations, as these tools form only part of a comprehensive assessment. Furthermore, they suggest that many analogous *black boxes* are found in modern medicine, such as magnetic resonance imaging (“MRI”), the results of which are routinely used by clinicians who cannot explain its inner workings without ethical or legal dilemma.

A clinician’s due diligence is tantamount to the quality of their decision-making process; for this reason, we root our analysis in interpreting the use of cAI through the perspective of decision analysis. One foundational concept in this, though commonly ignored, is that good decisions can and do lead to bad outcomes [56] and this is true for cAI and clinicians alike. We align with those who question the necessity of *explainability* of cAI asserting that cAI will not be implemented in a vacuum but in the context of all existing tools at the clinician’s disposal; as such cAI is to be used as a supplement to, rather than a substitute for, clinical decision making. A determination regarding a clinician’s culpability is one as to the reasonableness of their decision process and whether it met the standard of care. While the empirical performance of a *black box* is likely a compelling justification for its use, the use of cAI is not synonymous with concurrence, but rather with consultation with consideration for the entire clinical picture at hand. We find the comparison with MRI to be disanalogous, as the radiologists that interpret the imaging do understand the underlying physical mechanisms, even if the clinicians that subsequently make use of the radiologists’ reports do not; we feel the comparison of *black box* cAI with clinical practice guidelines to be more apt, given that approximately half of guideline recommendations are based on expert-opinion alone without supporting evidence [57, 58]. Clinical practice guidelines are routinely used to complement (not limit) medical decision-making when clinicians weigh the risks and benefits of recommendations in determining their suggested course of action; so too can *black box* cAI outputs supplement context and contribute to due diligence rather than detract from it. Whether or not *explainable*, clinicians are not only free to be critical of cAI output but are ethically and legally compelled to do so by leveraging the complete diagnostic context available to them.

### AI *Explainability* and Statute

**Table 10:** Discourse on AI *explainability* and legal statute

*Arguments*	*Rebuttals*
***IN FAVOUR*** ** of the necessity of** *** explainability*** ** of cAI**	
“A right of explanation was arguably first implemented in European General Data Protection Regulation (GDPR), and later adopted by some other jurisdictions.” [30] “Article 15 (1) [h] and Recital 71 of the General Data Protections Regulations of the European Union require businesses using personal data to explain how the program makes decisions and to provide data subjects with the right to ask why the model made the decision it did” [54]	“Since the right to explanation is contained only in the (non-binding) recital 71 of GDPR, there is an argument that a right to explanation of individual decisions does not derive from Art. 22(3) GDPR” [9]
***IN FAVOUR*** ** of the necessity of** *** explainability*** ** of cAI**	
“one can contest decisions, only on the basis of the ways, how the decision made; thus, without an explanation of how the algorithm works, it would be hard (if possible at all) to enforce a right to contest automated decisions and thus the rights to fair trial and effective remedy enshrined in Articles 6 and 13 of the European Convention on Human Rights.” [9] “Art. 3 para. 2a) [European Charter of Fundamental Rights requires] “free and informed consent” of the patient. This points to a “shared decision-making” by doctor and patient where the patient has the ultimate say. Medical AI can therefore only be used if patients have been informed about its essential functions beforehand—admittedly in an intelligible form. This makes it clear, however, that the European fundamental rights basically require the use of explainable AI in medicine (see also Art. 13 para. 1 of the proposed AI Act).” [59]	

Supporters of the necessity of *explainability* of cAI frequently identify portions of the European General Data Protection Regulation that in their view mandate *explainability* of all AI being developed with personal data, whereas critics stress that the critical wording relied upon for this opinion exists only in the non-binding recitals of the Regulation.

cAI statute is in its infancy globally with European regulations and guidelines seemingly the most developed in this sphere [60]; this lead to a predominantly Eurocentric legal perspective represented in the analyzed publications, though the US Food and Drug Administration was also mentioned, and the United States Federal Food, Drug, and Cosmetic Act by extension implicated. Interestingly, the American Office of Science and Technology Policy released recommendations calling for “explanations as to how and why a decision was made” (OSTP, 2022), and unambiguously demanding *explainability* (reasoning it necessary to correct errors and guard against harms), though this blueprint was not mentioned in the analyzed works. Most every author that touched on legal statute made mention of the European General Data Protection Regulation while only a few brought up the European Charter of Fundamental Rights [9, 59], yet interpreting the former remains rather elusive while the latter more compellingly demands *explainability* of cAI. Based on the arguments put forth we can only conclude that European fundamental rights preclude *autonomous black box* clinical decision making, though this is a mere subset of the possible implementations of *black box* cAI (such as human-in-the-loop [45] workflows wherein a clinician makes a diagnosis using all available tools including, but not limited to, a *black box*). Whether a general description of an algorithm’s inputs, performance and training data do not meet the definition of a cAI’s “essential functions” as suggested [59] remains to be judged.

### Achievability of AI *Explainability*

**Table 11:** Discourse on the achievability of AI *explainability*

*Arguments*	*Rebuttals*
***OPPOSED*** ** to the necessity of** *** explainability*** ** of cAI**	
“Explanations from current XAI methods superficially represent the computational complexity that underlies a prediction” [28] “Extracting information from models which may have millions of parameters and presenting this information in a way understandable to the human mind is an inherently reductive process” [8]	“it can be argued by analogy that if idealized scientific models such as the ideal gas law can provide genuine explanations that enable people to better understand complex natural phenomena, then XAI methods can provide genuine explanations too.” [23]
***OPPOSED*** ** to the necessity of** *** explainability*** ** of cAI**	
“An explanation that assumes a background in computer science, for instance, may be useful for the manufacturers and auditors of medical AI systems, but is likely to deliver next to no insight for a medical professional that lacks this technical background. Conversely, a simple explanation tailored to patients, who typically lack both medical and computer science backgrounds, is likely to provide little utility to a medical practitioner. […] post hoc explanation methods are not currently capable of meeting this challenge” [27]	“An explanation does not require knowing the flow of bits through an artificial intelligence system, no more than an explanation from humans requires knowing the flow of signals through human brain neurons” [9]

Advocates for *black box* cAI make the epistemic claim that *explainability* cannot be achieved in practice by virtue of the simplification that is intrinsically necessary of an explanation, and by the fact that different audiences require different explanations; those in favour of the necessity of *explainability* of cAI respond that simplified, idealized models, can provide generally accessible explanations of complex underlying processes.

We find the justifications provided for *black box* cAI insufficient. As an extension of Holm’s astute comment regarding the utility of idealized scientific models (2023) we contend that all medical science is in fact a simplified representation of complex natural phenomena that still provides genuine explanations. Though we concede that any quest for causal explanation can eventually be expounded to a level of inscrutability, perhaps put best by Feynman “the problem, you see, when you ask why something happens, how does a person answer why something happens?” [61], an explanation’s validity cannot be necessarily compromised by virtue of the inclusion of a simplification lest we accept that all medical science is similarly compromised. If this were the case then the entire discourse on clinician understanding and patient informed consent would be moot. While it is true that different audiences require different explanations tailored to the nature of their unique needs, the intended audience for cAI explanations is not ambiguous as suggested [27] and is arguably the clinician who can subsequently paraphrase and elaborate for the patient as needed; we would not question the intended audience of a consult note or lab result as it is clearly the referring physician, why should we expect anything different of *explainable* cAI? However, though *explainable* cAI is arguably achievable, this is only necessary but not sufficient grounds for establishing necessity.

### AI *Explainability* and Scientific Discovery

**Table 12:** Discourse on AI *explainability* and scientific discovery

*Arguments*	*Counter-arguments*
***IN FAVOUR*** ** of the necessity of** *** explainability*** ** of cAI**	
“correlations uncovered by XAI might turn out to be real but previously unknown biomedical relationships, in which case XAI could be used as a tool for scientific discovery” [28] “it has the potential to discover correlations that a human observer is totally ignorant of” [53]	“mechanistic explanations can lead to false conclusions, and mechanistic reasoning alone has been shown to have a high degree of fallibility. At times empirical results can be entirely contrary to mechanistic expectations, as in the case of prophylactic antiarrhythmic drugs actually acting to increase mortality from arrhythmia after recurrent acute myocardial infarction” [8] “Interpretability may thus feed a misguided expectation that understanding a set of associations valuable for specific diagnostic or prediction tasks will increase our ability to perform additional tasks to which those associations are not well suited and for which their accuracy has not been validated.[…] The long medical preference for radical mastectomy over less aggressive alternatives was driven by the pathophysiological theory that removing as much tissue from the breast as possible would reduce the probability of cancer recurrence. Only after a series of clinical trials was this theory shown to be false” [10]
***OPPOSED*** ** to the necessity of** *** explainability*** ** of cAI**	
“Ultimately, the primary goals of medicine are pragmatic: to relieve suffering and promote health. The elucidation of mechanisms comes secondary to this goal” [8]	

Those in favour of *explainable* cAI suggest that it may be used as a tool for scientific discovery, with explanations outlining previously unknown relationships within the data; critics point out that correlation does not imply causation and provide examples where false mechanistic reasoning has previously led to iatrogenic harm.

We feel that explanations from cAI tools may very well present previously unknown correlations or causations within the data, though outputs of *black boxes* can similarly be studied for input-output relationships. While efforts to chase down the conclusions of any cAI may turn out to be “misguided” [10] and fruitless on a case-by-case basis, such is the scientific method [62]. In any case, the primary purpose of cAI is not to fuel scientific discovery but to complement clinical care, and as such these considerations are tangential to the discussion of the necessity of *explainability* thereof.

## Conclusion

While the literature remains divided on the subject, the arguments put forth to date do not necessitate *explainability* from clinical implementations of AI. The issues raised regarding fundamental rights legislation and the biomedical ethical principle of justice [19] in the context of procedural fairness compellingly preclude the use of autonomous *black boxes*, but are not convincing regarding human-in-the-loop [45] implementations. With or without explanations for its outputs, cAI can be critically appraised as required by evidence-based medicine practices in a fashion similar to that used for existing empirical data.

The literature appropriately highlights specific instances in medicine where empirical approaches are employed in the absence of mechanistic understanding, such as the use of lithium as a medication. However, this reliance on empiricism is far more prevalent than these discrete examples imply, with estimates suggesting that up to two-thirds of patients receive no biomedical explanation for at least one of their symptoms [63], resulting in so-called idiopathic diagnoses. Another common medical practice, providing a diagnosis upon the exclusion of all other possibilities within the differential, by definition uses no specific mechanistic knowledge of the assumed disease. Thus, patients and clinicians already routinely operate without mechanistic understanding and rely on empirical practices.

Clinical practice guidelines are universally applied despite approximately half of their recommendations being unsupported by direct evidence [57, 58], effectively making them *black boxes* in their own right. Clinicians are not forced to dogmatically follow the outputs of *black box* cAI [31] any more than they are automatons algorithmically bound to clinical practice guidelines at present.

From the perspective of mathematical optimization, it is clear that algorithms with explainability inherently incur a non-negative performance cost compared to those without this requirement. Although this issue is debated in the literature, the need for explainability effectively prioritizes the value of explanation over performance. Clinicians’ trust in cAI ought to be predicated on the quality of the AI training and performance, which are elucidated through development transparency and not algorithm *explainability*. In turn, patients trust clinicians by virtue of their sound decision-making processes, which ought to incorporate cAI, be it *black box* or with *explainability*, into the clinical picture painted by all information and tools available to the clinician. Arguments against *explainability* speaking to lack of achievability are practically irrelevant. Concerns of *black box* cAI contributions to systemic inequality by virtue of data availability, bias, and prejudice are not unfounded, but apply equally to cAI possessing *explainability* as these are functions of the training data and development process. Notably, some arguments in the literature in favour of the necessity of cAI *explainability* problematically conflate *black box* AI use with automated decision making, or similarly equate the use of cAI with the exclusive use of cAI.

## Data Availability

No datasets were generated or analysed during the current study.

## Appendix: Works Included in this Systematized Review, after Retrieval and Screening

Adams J. (2023). Defending explicability as a principle for the ethics of artificial intelligence in medicine. *Med Health Care Philos*, 26(4), 615–623. 10.1007/s11019-023-10175-7

Amann J, Blasimme A, Vayena E, Frey D, & Madai VI. (2020). Explainability for artificial intelligence in healthcare: A multidisciplinary perspective. *BMC Medical Informatics and Decision Making*, *20*(1), 310. 10.1186/s12911-020-01332-6

Aranovich, T., & Matulionyte, R. (2023). Ensuring AI explainability in healthcare: Problems and possible policy solutions. *Information & Communications Technology Law*, *32*(2), 259–275. 10.1080/13600834.2022.2146395

Astromske, K., Peicius, E., & Astromskis, P. (2021). Ethical and legal challenges of informed consent applying artificial intelligence in medical diagnostic consultations. *AI & Society*, *36*(2), 509–520. 10.1007/s00146-020-01008-9

Banja JD, Hollstein RD, & Bruno MA. (2022). When Artificial Intelligence Models Surpass Physician Performance: Medical Malpractice Liability in an Era of Advanced Artificial Intelligence. *J Am Coll Radiol*, *19*(7), 816–820. 10.1016/j.jacr.2021.11.014

Bradshaw T.J., McCradden M.D., Jha A.K., Dutta J., Saboury B., Siegel E.L., & Rahmim A. (2023). Artificial Intelligence Algorithms Need to Be Explainable-or Do They? *Journal of Nuclear Medicine*, *64*(6), 976–977. 10.2967/jnumed.122.264949

Chan, B. (2023). Black-box assisted medical decisions: AI power vs. Ethical physician care. *Medicine Health Care and Philosophy*, *26*(3), 285–292. 10.1007/s11019-023-10153-z

Da Silva, M. (2023). Explainability, Public Reason, and Medical Artificial Intelligence. Ethical *Theory and Moral Practice*, *26*(5), 743–762. 10.1007/s10677-023-10390-4

Durán, J. M., & Jongsma, K. R. (2021). Who is afraid of black box algorithms? On the epistemological and ethical basis of trust in medical AI. Journal of Medical Ethics, medethics-2020-106820. 10.1136/medethics-2020-106820

Friedrich, A., Mason, J., & Malone, J. (2022). Rethinking explainability: Toward a postphenomenology of black-box artificial intelligence in medicine. Ethics and Information Technology, 24(1). 10.1007/s10676-022-09631-4

Grote, T. (2023). The Allure of Simplicity: On Interpretable Machine Learning Models in Healthcare. *Philosophy of Medicine*, 4(1), Article 1. 10.5195/pom.2023.139

Hacker, P., Krestel, R., Grundmann, S., & Naumann, F. (2020). Explainable AI under contract and tort law: Legal incentives and technical challenges. *Artificial Intelligence and Law*, 28(4), 415–439. 10.1007/s10506-020-09260-6

Hatherley, J., Sparrow, R., & Howard, M. (2022). The Virtues of Interpretable Medical Artificial Intelligence. *Cambridge Quarterly of Healthcare Ethics*. 10.1017/S0963180122000305

Heinrichs B & Eickhoff SB. (2020). Your evidence? Machine learning algorithms for medical diagnosis and prediction. *Hum Brain Mapp*, 41(6), 1435–1444. 10.1002/hbm.24886

Herington J., McCradden M.D., Creel K., Boellaard R., Jones E.C., Jha A.K., Rahmim A., Scott P.J.H., Sunderland J.J., Wahl R.L., Zuehlsdorff S., & Saboury B. (2023). Ethical Considerations for Artificial Intelligence in Medical Imaging: Deployment and Governance. *Journal of Nuclear Medicine*, *64*(10), 1509–1515. 10.2967/jnumed.123.266110

Herrmann, M., Wabro, A., & Winkler, E. (2024). Percentages and reasons: AI explainability and ultimate human responsibility within the medical field. *Ethics and Information Technology*, *26*(2). 10.1007/s10676-024-09764-8

Holm, S. (2023). On the Justified Use of AI Decision Support in Evidence-Based Medicine: Validity, Explainability, and Responsibility. *Cambridge Quarterly of Healthcare Ethics*. 10.1017/S0963180123000294

Kempt, H., Heilinger, J., & Nagel, S. (2022). Relative explainability and double standards in medical decision-making Should medical AI be subjected to higher standards in medical decision-making than doctors? *Ethics and Information Technology 24*(2). 10.1007/s10676-022-09646-x

London, A. J. (2019). Artificial Intelligence and Black-Box Medical Decisions: Accuracy versus Explainability. *Hastings Center Report*, *49*(1), 15–21. 10.1002/hast.973

Matulionyte, R., Nolan, P., Magrabi, F., & Beheshti, A. (2022). Should AI-enabled medical devices be explainable? *International Journal of Law and Information Technology*, *30*(2), 151–180. 10.1093/ijlit/eaac015

McCoy, L., Brenna, C., Chen, S., Vold, K., & Das, S. (2022). Believing in black boxes: Machine learning for healthcare does not need explainability to be evidence-based. *Journal of Clinical Epidemiology*, *142*, 252–257. 10.1016/j.jclinepi.2021.11.001

Nimnuan, C., Hotopf, M., & Wessely, S. (2001). Medically unexplained symptoms: An epidemiological study in seven specialities. *Journal of Psychosomatic Research*, 51(1), 361–367. 10.1016/S0022-3999(01)00223-9

Ochoa, J., & Marquardt, A. (2023). Editorial: Transparent machine learning in bio-medicine. *Frontiers in Bioinformatics, 3*. 10.3389/fbinf.2023.1264803

Onitiu, D. (2023). The limits of explainability & human oversight in the EU Commission’s proposal for the Regulation on Al-a critical approach focusing on medical diagnostic systems. *Information & Communications Technology Law*, *32*(2), 170–188. 10.1080/13600834.2022.2116354

Office of Science and Technology Policy (2022). Blueprint for an AI Bill of Rights: Making automated systems work for the American people. *The White House*. https://www.whitehouse.gov/ostp/ai-bill-of-rights/

Pierce, R., Van Biesen, W., Van Cauwenberge, D., Decruyenaere, J., & Sterckx, S. (2022). Explainability in medicine in an era of AI-based clinical decision support systems. *Frontiers in Genetics*, *13*. 10.3389/fgene.2022.903600

Rueda, J., Rodríguez, J. D., Jounou, I. P., Hortal-Carmona, J., Ausín, T., & Rodríguez-Arias, D. (2022). “Just” accuracy? Procedural fairness demands explainability in AI-based medical resource allocations. *AI & Society*, 1–12. 10.1007/s00146-022-01614-9

Sendak, M., Elish, M. C., Gao, M., Futoma, J., Ratliff, W., Nichols, M., Bedoya, A., Balu, S., & O’Brien, C. (2020). “The human body is a black box”: Supporting clinical decision-making with deep learning. *Proceedings of the 2020 Conference on Fairness, Accountability, and Transparency*, 99–109. 10.1145/3351095.3372827

Stöger, K., Schneeberger, D., & Holzinger, A. (2021). Medical Artificial Intelligence: The European Legal Perspective. *Communications of the ACM*, *64*(11), 34–36. 10.1145/3458652

Theunissen, M., & Browning, J. (2022). Putting explainable AI in context: Institutional explanations for medical AI. *Ethics and Information Technology*, *24*(2). 10.1007/s10676-022-09649-8

Ursin, F., Lindner, F., Ropinski, T., Salloch, S., & Timmermann, C. (2023). Levels of explicability for medical artificial intelligence: What do we normatively need and what can we technically reach? *Ethik in der Medizin*, *35*(2), 173–199. 10.1007/s00481-023-00761-x

Ursin F, Timmermann C, & Steger F. (2022). Explicability of artificial intelligence in radiology: Is a fifth bioethical principle conceptually necessary? *Bioethics*, *36*(2), 143–153. 10.1111/bioe.12918

Van Der Veer S.N., Riste L., Cheraghi-Sohi S., Phipps D.L., Tully M.P., Bozentko K., Atwood S., Hubbard A., Wiper C., Oswald M., & Peek N. (2021). Trading off accuracy and explainability in AI decision-making: Findings from 2 citizens’ juries. *Journal of the American Medical Informatics Association*, *28*(10), 2128–2138. 10.1093/jamia/ocab127

Wadden, J. (2021). What Kind of Artificial Intelligence Should We Want for Use in Healthcare Decision-Making Applications? *Canadian Journal of Biothics-Revue Canadienne de Bioethique*, *4*(1), 94–100.

Wadden, J. (2022). Defining the undefinable: The black box problem in healthcare artificial intelligence. *Journal of Medical Ethics*, *48*(10), 764–768. 10.1136/medethics-2021-107529

Yoon, C., Torrance, R., & Scheinerman, N. (2022). Machine learning in medicine: Should the pursuit of enhanced interpretability be abandoned? *Journal of Medical Ethics*, *48*(9), 581–585. 10.1136/medethics-2020-107102

Yu, F., Moehring, A., Banerjee, O., Salz, T., Agarwal, N., & Rajpurkar, P. (2024). Heterogeneity and predictors of the effects of AI assistance on radiologists. *Nature Medicine*, 30(3), 837–849. 10.1038/s41591-024-02850-w
